# P-1611. Clinical Evaluation of the Selux Rapid Next Generation Phenotypic Antimicrobial Susceptibility System for both Direct-from-Colony and Positive Blood Cultures

**DOI:** 10.1093/ofid/ofae631.1778

**Published:** 2025-01-29

**Authors:** Margie A Morgan, Stuart G Smith, Deisy Contreras

**Affiliations:** Cedars-Sinai Medical Center, Los Angeles, California; Cedars-Sinai Medical Center, Los Angeles, California; Cedars-Sinai Medical Center, Los Angeles, California

## Abstract

**Background:**

The Selux next-generation phenotyping (NGP) system (SeluxDx) utilizes a proprietary 384-well panel for Gram-positive and Gram-negative antibiotic susceptibility testing available direct-from-colony and Gram-negative from positive blood culture.

**Methods:**

Previously tested bacteria on the Phoenix M50 were used for direct-from-colony assessment. Gram-negative (n=100) and Gram-positive (n=30), were grown on 5% sheep’s blood agar plates and incubated overnight at 35^o^C. Testing dilution was prepared in saline in the range of 0.4-0.6 McFarland and then inoculated into either the Gram-positive or Gram-negative panel utilizing the system’s automated inoculator platform. Positive blood cultures were concentrated using the Selux Separator automated platform and a pre-prepared McFarland sample was generated to inoculate the Gram-negative panel, and a viable bacterial pellet produced for identification testing. All panels were processed using the Selux Analyzer platform and categorical and essential agreement was calculated for FDA approved bug-drug combinations.

**Results:**

The overall essential agreement for the Gram-negative panel was 97% (97/100) and categorical agreement was 94% (94/100). Two very major errors included Ertapenem in an *Escherichia coli* and an *Enterobacter cloacae complex* isolate where the Selux system failed to detect resistance. For the Gram-positive panel, categorical agreement was found to be 96.7% (29/30) with one very major error in *Staphylococcus aureus* for Clindamycin. Selux generated an AST result for 98% (40/41) of the concentrated blood culture samples. The overall categorical agreement between Selux and the Accelerate Pheno® system was 93%. Major discrepancies (2.4%) and very major discrepancies (4.8%) detected involved the testing of Piperacillin/Tazobactam. The pellet generated an identification to species on 45/48 (94%) of samples using the Bruker MALDI Biotyper®.

**Conclusion:**

The Selux NPG system proved to be a reliable rapid automated platform for both Gram-negative and Gram-positive isolates from direct-from-culture and for rapid AST from positive blood culture Gram-negative isolates. The 384-well panel allows for future adaption of novel antimicrobial agents to meet the demand of an ever-changing resistance landscape.

**Disclosures:**

**All Authors**: No reported disclosures

